# Adenosine Receptor A_2a_, but Not A_1_ in the rVLM Participates Along With Opioids in Acupuncture-Mediated Inhibition of Excitatory Cardiovascular Reflexes

**DOI:** 10.3389/fnins.2019.01049

**Published:** 2019-10-04

**Authors:** Shaista Malik, Tracy Samaniego, Zhi-Ling Guo

**Affiliations:** Department of Medicine, Susan Samueli Integrative Health Institute, University of California, Irvine, Irvine, CA, United States

**Keywords:** blood pressure, adenosine, opioids, brain stem, acupuncture

## Abstract

Electroacupuncture (EA) can be used to lower high blood pressure (BP) in clinical practice. However, precise mechanisms underlying its effects on elevated BP remain unclear. Our previous studies have shown that EA at the P5-6 acupoints, overlying the median nerve, attenuates elevated BP induced by gastric distension (GD) through influence on rostral ventrolateral medulla (rVLM). Although adenosine is released during neuronal activation in the rVLM, its role in acupuncture-cardiovascular regulation is unknown. The purinergic system is involved in cardiovascular pressor and depressor responses, including via selective activation of A_1_ and A_2__a_ rVLM receptors, respectively. The action of A_2__a_ receptor stimulation in the central nervous system may be further regulated through an endogenous opioid mechanism. However, it is uncertain whether this putative action occurs in the rVLM. We hypothesized that adenosine in the rVLM contributes to EA modulation of sympathoexcitatory reflexes through an A_2__a_ but not an A_1_ adenosine receptor-opioid mechanism. EA or sham-EA was applied at the P5-6 acupoints in Sprague-Dawley male rats subjected to repeated GD under anesthesia. We found that EA (*n* = 6) but not sham-EA (*n* = 5) at P5-6 significantly (*P* < 0.05) attenuated GD-induced elevations in BP. EA modulation of sympathoexcitatory cardiovascular reflexes was reversed significantly after rVLM microinjection (50 nl) of 8-SPT (10 mM; non-selective adenosine receptor antagonist; *n* = 7) or SCH 58261 (1 mM; A_2__a_ receptor antagonist; *n* = 8; both *P* < 0.05), but not by DPCPX (3 mM; A_1_ receptor antagonist; *n* = 6) or the vehicle (5% dimethylsulfoxide; *n* = 6). Moreover, microinjection of an A_2__a_ receptor agonist, CGS-21680 (0.4 mM; *n* = 8) into the rVLM attenuated GD-induced pressor responses without EA, which mimicked EA’s inhibitory effects (*P* < 0.05). After blockade of opioid receptors with naloxone (1 mM) in the rVLM, SCH 58261’s reversal of EA’s effect on GD-induced pressor responses was blunted, and CGS-21680-mediated inhibitory effect on pressor responses was not observed. Furthermore, neurons labeled with adenosine A_2__a_ receptors were anatomically co-localized with neurons stained with enkephalin in the rVLM. These data suggest that the involvement of rVLM adenosine A_2__a_ receptors in EA modulation of GD-induced pressor reflexes is, at least in part, dependent on the presence of endogenous opioids.

## Introduction

Acupuncture has been practiced for at least three millennia in Asia, and it increasingly is accepted as a potential therapy for many disorders in the Western world, including hypertension (Andersson, 1993; Li and Longhurst, 2010; Li et al., 2015). In this respect, acupuncture offers a non-pharmacological as well as an adjunctive approach to reduce high BP with fewer adverse effects compared to anti-hypertensive drugs. However, specific mechanisms underlying the physiological effects of acupuncture remain unclear, and the postulated actions of acupuncture have not been examined by rigorous scientific studies.

There is clear evidence that the CNS plays a critical role in the development and maintenance of hypertension. Increase in the activity of the sympathetic nervous system contributes to high BP. Our previous studies have shown that EA attenuates reflex elevation in BP induced by visceral stimulation, like GD (Li et al., 2002). The rVLM is a brain nucleus that critically controls sympathetic outflow and cardiovascular function (Dampney, 1994). Several modulatory neurotransmitters, including endogenous opioids and GABA in the rVLM, contribute to central processing of somatic afferent nerve stimulation induced by EA, resulting in improvement of elevated BP (Tjen-A-Looi et al., 2007; Zhou et al., 2007). However, inhibiting the action of any single neurotransmitter in the rVLM does not reverse EA’s hypotensive effect completely, implying the involvement of other neuromodulators during EA. In this regard, adenosine, which is released during neuronal activation (Spyer et al., 1997; Ribeiro et al., 2002), is another potential neuromodulator of EA’s effect on elevated BP. The purinergic system primarily modulates neuronal function through P1 (subclassified into adenosine A_1_, A_2__a_, A_2__b_, and A_3_) receptors (Ribeiro et al., 2002; Borea et al., 2018). Selective activation of A_1_ or A_2__a_ receptors in the rVLM induces pressor or depressor responses, respectively (Nassar and bdel-Rahman, 2008, 2009; Jiang et al., 2011) and this is consistent with adenosine A_1_ and A_2__a_ receptors generally having opposing actions at cellular and neuronal levels (Ribeiro et al., 2002; Borea et al., 2018). It is unknown if A_2__a_ or A_1_ receptors in the rVLM contribute to EA’s modulation of sympathoexcitatory reflexes.

The action of A_2__a_ receptors in the CNS may be regulated, at least in part, through an opioid mechanism (Ribeiro et al., 2002; By et al., 2011). Our previous studies have shown that EA lowers reflex elevations in BP through an opioid mechanism, modulating rVLM activity (Tjen-A-Looi et al., 2007). It remains unknown if opioid peptides are involved in adenosine regulation of BP, especially during EA. In this study, we evaluated the role of A_1_ or A_2__a_ receptors in the rVLM in modulating EA’s action in lowering elevated BP as well as its potential relevance to endogenous opioids. We hypothesized that A_2__a_ but not A_1_ adenosine receptors in the rVLM contributes to EA modulation of sympathoexcitatory reflexes, and the action of A_2__a_ adenosine receptors is associated with the presence of opioids in the rVLM.

## Materials and Methods

### Anesthesia and Surgical Preparations

Studies were performed on adult Sprague-Dawley male rats (450–500 g) following an overnight fast. All experimental protocols and preparations were reviewed and approved by the Animal Care and Use Committee of the University of California at Irvine. Also, the study conformed to the American Physiological Society’s Guiding Principles for Research Involving Animals.

Anesthesia was induced with ketamine (100 mg/kg, im) and maintained with α-chloralose (50–60 mg/kg, iv). Additional doses of α-chloralose (25–30 mg/kg, iv) were given as necessary to maintain an adequate depth of anesthesia by observing the absence of conjunctival reflex response. The femoral vein was cannulated for the administration of fluids, and the femoral artery was cannulated and connected to a pressure transducer (Statham P23 ID, Gould) to monitor systemic BP. Heart rate was derived from the pulsatile pressure waveform signal. The trachea was intubated and respiration was maintained with a ventilator (model 661, Harvard Apparatus, Holliston, MA, United States). Arterial blood gases and pH were measured periodically with a blood-gas analyzer (model ABL5, Radiometer, Copenhagen, Denmark) and were kept within normal physiological limits (pH 7.35 – 7.43, PO2 > 100 mmHg and PCO2 30–40 mmHg) by adjusting the ventilation rate or volume, enriching the inspired oxygen supply and infusion of a solution of 8% sodium bicarbonate at a rate of 0.1 ml/min. Body temperature was monitored with a rectal thermometer (model 44TD) and maintained between 36.0 and 37.5°C with a heating pad and lamp.

### Induction of Pressor Reflexes

Consistent reflex increases in BP were induced by GD, as we described in detail previously (Li et al., 2002; Zhou et al., 2005a; Guo et al., 2018). Briefly, an unstressed 2-cm diameter latex balloon (catalog no.: 391766)^1^ was attached to a polyurethane tube (3-mm diameter) and inserted into the stomach through the mouth and esophagus. Transmural pressure was determined by measuring the pressure required to inflate the balloon with various volumes of air before it was inserted into the stomach (Li et al., 2002). The balloon was palpated manually from the surface of the body during insertion, as it was passed through the esophagus into the stomach to confirm the positioning of the balloon inside the stomach. A syringe was attached to the cannula to inflate and deflate the balloon with air, while a manometer through a T-connection was used to monitor balloon pressure. Distention pressures were selected to fall within the range that a rat normally experiences during ingestion of food and fluids in a single meal (Li et al., 2002; Guo et al., 2018). To induce increases in BP, the balloon was inflated inside the stomach. Increases in BP were observed within 30 s of inflation. The balloon was deflated within 30 s after reaching the maximal rise in BP. We did not include animals in the study when the balloon was verified post mortem to be in the esophagus. This animal model has been used in several previous studies to assess acupuncture’s sympatholytic action (Li et al., 2002, 2013; Crisostomo et al., 2005; Guo et al., 2018).

### Acupuncture Application

**FIGURE 1:**
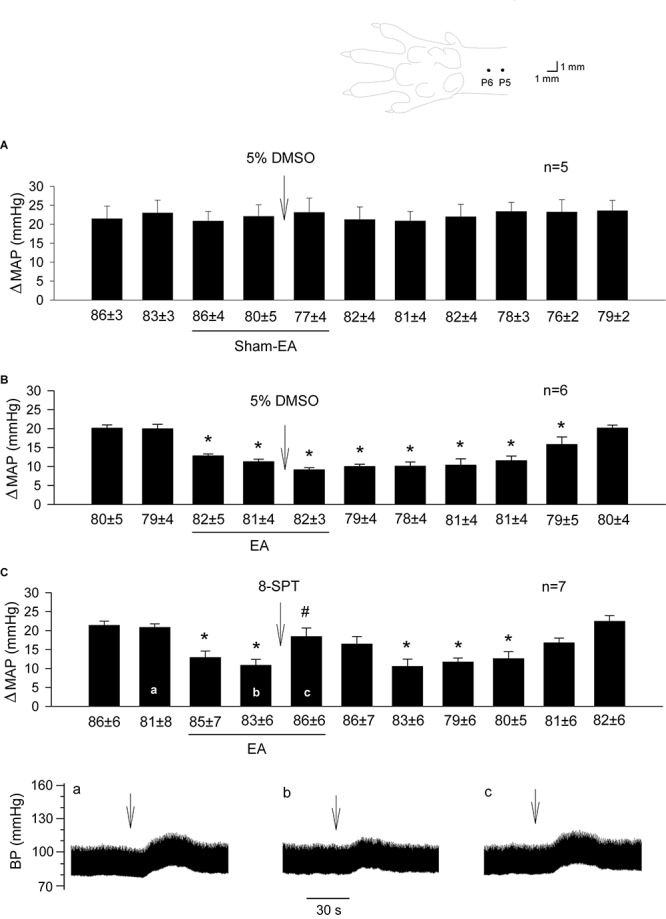
Influence of blockade of adenosine receptors in the rVLM on EA modulation of GD-induced pressor reflexes. The diagram above bar graphs displays the sites of the P5 and P6 acupoints (Hua, 1994). Bars represent increases in mean arterial blood pressure (MAP) following GD. Values below each bar indicate the baseline MAP (means ± SE) before GD. **(A,B)** 5% DMSO (50 nl) was injected into the rVLM as sham-EA **(A)** or EA **(B)** was conducted at P5-6; **(C)** microinjection of 8-SPT (10 mM in 50 nl; a non-selective adenosine receptor antagonist) into the rVLM with EA treatment. ^∗^*P* < 0.05, a decrease of GD response after the onset of EA; ^#^*P* < 0.05, after vs. before microinjection into the rVLM. Labels a-c in **(C)** indicate examples of the original BP tracings of a rat; ↓, time of GD application. These data suggest that adenosine in the rVLM is involved in EA modulation of GD-induced pressor reflexes.

P5 and P6 acupoints are located on forelimbs correspondingly 2.5 and 4.0 mm above the flexor crease in the paw (Figure 1), between the tendons of the palmaris longus and flexor carpi radialis muscles overlying the median nerve (Hua, 1994). Acupuncture needles (40 gauge stainless steel; diameter, 0.16 mm) were inserted (through the skin) into P5-6 acupoints bilaterally at a depth of ∼3 mm. Stimulation of these acupoints has been shown to lower BP during sympathoexcitatory reflexes (Li et al., 2002; Guo et al., 2018) and in chronic hypertension (Li et al., 2015). The needles were connected to a photoelectric stimulus isolation unit and the stimulator (model no. S88, Grass, West Warwick, RI, United States). Each set of electrodes was stimulated separately as a positive and negative pole, so that current did not flow from one location to the contralateral forelimb. Correct placement of the needles at the P5-P6 acupoints was confirmed by observing slight repetitive paw twitches at or near motor threshold during EA. The twitches were important observations to confirm the stimulation of motor fibers in the median nerve. Gallamine triethiodide (4 mg/kg) was administered intravenously before application of 30 min EA (2 Hz, 0.5 ms, 0.3–0.5 mA) to avoid muscle movement during stimulation of the median nerve. We typically lowered current during electrical stimulation to a level just below the motor threshold. The frequency (2 Hz) for EA was used to modulate reflex-induced elevations in BP as we have demonstrated previously (Li et al., 2002, 2013; Guo et al., 2018). During sham-EA, needles were inserted into the same acupoints without subsequent electrical stimulation, which was an appropriate control of the acupuncture study (Li et al., 2002, 2013; Tjen-A-Looi et al., 2004; Guo et al., 2018).

### Microinjection Into the rVLM

Animals were placed in a stereotaxic head frame to position their heads with the floor of the fourth ventricle in a horizontal position. A partial craniotomy was performed to expose the medulla to allow access to the rVLM. We performed microinjections using a modified CMA microdialysis probe that was 14 mm long (tip diameter 0.24 mm; CMA Microdialysis, Stockholm, Sweden) and lacked the microdialysis membrane. After being positioned by a micromanipulator (Kopf Instruments), the probe was inserted unilaterally (side chosen randomly) into the medulla with visual approximation at a 90° angle relative to the dorsal surface of the medulla, 1.8 – 2.3 mm lateral from the midline, 1.1 – 1.6 mm rostral to the obex, and advanced 3.0 – 3.3 mm from dorsal toward the ventral surface. These coordinates provide access to a region in the rVLM that has been found to contain premotor sympathoexcitatory cells (Li et al., 2002, 2013). Proper positioning of probes in the rVLM was confirmed by noting a 5–10 mmHg elevation in BP following the probe insertion as well as microinjection of glutamate (2 nmol in 50 nl) (Guo et al., 2009). The correct location of the rVLM was confirmed further by histological examination of stain from 0.5% pontamine sky blue, which was injected along with the chemicals tested after each experiment (Guo et al., 2009). The probe was connected to a microsyringe fastened to microdialysis pump (CMA/102, North Chelmsford, MA, United States) through a fluorinated ethylene propylene tubing (0.12-mm inner diameter) and tubing adaptors. The injection of the low possible volume of 50 nl was carried out at a rate of 0.6 μl/min over a 5-s period. Of note, we have demonstrated significant blockade of EA’s actions following unilateral administration of drugs, which allows maintaining optimal physiological state to study EA-modulation of cardiovascular responses. This simplified approach of unilateral microinjection has been proven to be successful (Li et al., 2002; Crisostomo et al., 2005; Tjen-A-Looi et al., 2007). Microinjection of the vehicle into the rVLM and drug into surrounding regions provided chemical and anatomical controls. We allowed any small transient changes in basal BP induced by injecting drugs to recover before conducting an experiment (Li et al., 2002, 2013; Tjen-A-Looi et al., 2007).

### Drugs

The following drugs were used: a non-selective opioid receptor antagonist, naloxone (1 mM) (Li et al., 2002); adenosine A_2__a_ receptor agonist, CGS-21680 (0.4 mM) (Ichinose et al., 2009); adenosine receptor antagonists: non-selective 8-(*p*-sulfophenyl)theophylline (8-SPT, 10 mM) (Ichinose et al., 2009); A_1_ selective, 1,3-Dipropyl-8-cyclopentylxanthine (DPCPX, 3 mM) (Collis et al., 1989; Nassar and bdel-Rahman, 2009); A_2__a_ selective, SCH 58261 (1 mM) (Jiang et al., 2011). The vehicles for these drugs are normal saline or 5% dimethylsulfoxide (DMSO). All chemicals were purchased from Sigma Aldrich (St. Louis, MO, United States). Affinities, specificities, and dosages of the drugs are documented in the previous studies, as indicated by cited references for each individual one mentioned above. The solution of each drug or vehicle contained 0.5% pontamine sky blue for histological examination of the injection site after the experiment.

### Experimental Protocols

#### Role of Adenosine Receptors in Acupuncture Inhibition of Reflex Elevations in BP

A 30-min stabilization period was allowed after the surgical procedure. Rats were subjected to 11-repeated GDs every 10 min, and BPs were recorded and measured before and after each GD, as described in the methods section. Ten-minute intervals between GDs prevented tachyphylaxis of the cardiovascular responses (Li et al., 2002; Guo et al., 2018). In each experiment, after recording two repeatable elevations in BP in response to GDs, EA started right after the 2nd time of GD and ended after the 5th time of GD. EA was conducted for 30 min during which three GDs (e.g., 3rd – 5th times of GD) were performed. We applied EA at P5-P6 bilaterally at a frequency of 2 Hz (0.3–0.5 mA, 0.5-ms duration). A non-selective adenosine receptor antagonist, 8-PST or its vehicle (5% DMSO) was microinjected into the rVLM 5 min before the 5th time of GD and the termination of EA. Afterward, six times of GDs were conducted. In rats treated with sham-EA, the same procedure was carried out as that performed in the EA-treated animal, except for that the acupuncture needles were inserted bilaterally into P5-6 acupoints without electrical stimulation. The 5% DMSO was microinjected into the rVLM 5 min before the end of 30 min of sham-EA.

#### Specificity of Adenosine A_1_ and A_2__a_ Receptors in Acupuncture Inhibition of Reflex Elevations in BP

Similar to the protocol used to examine the effect of 8-PST on EA’s action in the rVLM described above, SCH 58261 (1 mM, A_2__a_ receptor-selective antagonist) or DPCPX (3 mM, A_1_ receptor-selective antagonist) were microinjected unilaterally into the rVLM 5 min before termination of 30 min of EA. BP responses to GD in the rat were measured every 10 min before, during, and for 60 min after EA. The vehicle for both drugs was 5% DMSO, similar to that for the 8-PST.

#### Influence of Activation or Inhibition of A_2__a_ Receptors in the rVLM on Pressor Reflexes

In separate animals without treatment with EA or sham-EA, six-repeated GDs were carried out every 10 min. Five min after the establishment of two consistent pressor responses to GD, we performed unilateral microinjection (50 nl) of 5% DMSO, SCH 58261 (1 mM; adenosine A_2__a_ receptor-specific antagonist) or CGS-21680 (0.4 mM; adenosine A_2__a_ receptor-specific agonist) into the rVLM. Five min later, repeated GDs were conducted for four times, lasting for 40 min.

#### Contribution of Endogenous Opioids to Adenosine A_2__a_ Receptor-Mediated Acupuncture Modulation of Pressor Responses

In separate rats that underwent EA treatment, we measured BP responses to 11-repeated GDs every 10 min. EA at P5-P6 was employed for 30 min following two repeatable responses to GDs, similar to the protocol described above. Differently, in this protocol, 5 min before termination of EA or the 5th time of GD, naloxone (1 mM; a non-selective opioid receptor antagonist), or 0.9% normal saline (the vehicle for naloxone) were administered (50 nl) unilaterally into the rVLM. Five min after the end of EA or the 6th time of GD, we microinjected (50 nl) SCH 58261 (1 mM, adenosine A_2__a_ receptor-specific antagonist), CGS-21680 (0.4 mM, adenosine A_2__a_ receptor-specific agonist) or 5% DMSO into the same site of the rVLM. Afterward, GDs were conducted repeatedly for five times.

### Histology

Rats were euthanized under deep anesthesia with additional α-chloralose, followed by saturated potassium chloride (1 M) at the end of each experiment. The stomach was exposed to confirm the placement of the balloon. The medulla oblongata was removed and submerged in 4% paraformaldehyde for at least 72 h. Frozen 60-μm coronal sections were cut with a cryostat microtome (Leica CM1850 Heidelberger Strasse, Nussloch, Germany) to confirm the microinjection sites histologically. Blue dye spots were identified with a microscope. Using the atlas of Paxinos and Watson as a guide (Paxinos and Watson, 2005), places of microinjections in the medulla were plotted with Corel Presentation software on reconstructed coronal sections (Li et al., 2002, 2013).

### Immunohistochemistry

Adult male Sprague-Dawley rats were anesthetized with a large dose of ketamine/xylazine (0.6–1.0 ml, im). Transcardial perfusion was performed using 300 ml of 0.9% saline solution followed by 300 ml of 4% paraformaldehyde in 0.1 M phosphate buffer (pH 7.4). Medulla oblongata was harvested and sliced into 30 μm sections using a cryostat microtome (Leica CM1850). Medullary sections were collected serially.

Free-floating sections were used for immunohistochemical staining. Briefly, after washing for 30 min (10 min × 3 times) using phosphate-buffered saline containing 0.3% Triton X-100 (PBST; pH = 7.4), brain sections were treated for 1 h with 1% normal donkey serum (Jackson Immunoresearch Laboratories, West Grove, PA, United States). The sections were incubated with a primary rabbit anti-adenosine A_2__a_ receptor antibody (1:200; Abcam, Cambridge, MA, United States) and a mouse anti-enkephalin antibody (1:200; EMD Millipore Corporation, Temecula, CA, United States) at 4°C for 48 h. The tissues subsequently were rinsed three times (10 min for each rinse) in PBST and incubated with a fluorescein-conjugated donkey anti-rabbit and a rhodamine-conjugated donkey anti-mouse (1:200 dilution; Jackson Immunoresearch Laboratories) for 24 h at 4°C. These secondary antibodies raised in the donkey are made for multiple labels. They have minimal cross-reactivity to other non-specific species (2010 catalog specializing in secondary antibodies; Jackson Immunoresearch Laboratories). Brain sections on the slide were air-dried. The slides were coverslipped using a mounting medium (Vector Laboratories, Burlingame, CA, United States). In immunohistochemical control studies, no labeling was detected when the primary or secondary antibody was omitted.

Image data analysis: Brain sections were scanned and examined with a standard fluorescent microscope (Nikon, E400, Melville, NY, United States). Two epifluorescence filters (B-2A, or G-2A) equipped in a fluorescent microscope were used to identify single stains appearing as green (fluorescein) or red (rhodamine) in brain sections. Immunoreactive neurons labeled with adenosine A_2__a_ receptors and enkephalin appeared as bright green and red colors, respectively (Figure 6). Selected sections were evaluated further using a Zeiss (LSM 780) laser scanning confocal microscope (Zeiss LSM 710, Meta system; Zeiss, Thornwood, NY, United States). This apparatus was equipped with HeNe and Argon lasers and allowed operation of multiple channels. Lasers of 488- and 543-nm wavelengths were used to excite fluorescein and rhodamine. Each confocal section analyzed was limited to a 0.5-μm thickness in the Z-plane. Digital images of the labels were captured and analyzed with software (Zeiss LSM) provided with this microscope. Images in two colors in the same plane were merged to reveal the relationship between two labels. Single- and double-labeled neurons were evaluated.

### Statistical Analyses

Reflex responses are expressed as the difference in MAP comparing steady-state baseline BP and pressure at peak response. Changes in MAP are presented as bar histograms. Data are presented as means ± SE. The increases in BPs before and after delivery of experimental drug or vehicle were compared by a one-way repeated-measures ANOVA followed *post hoc* by the Student–Newman–Keuls test. Data are plotted and analyzed with the Kolmogorov–Smirnov test for normal data distribution and normalized when necessary with SigmaPlot (Jandel Scientific Software, San Rafael, CA, United States). All statistical analyses were performed with SigmaPlot/Stat (Jandel Scientific). Values were considered to be significantly different when *P* < 0.05.

## Results

### The Action of Adenosine Receptors in EA Inhibition of Reflex Elevations in BP

BP responses to GD were consistent in a group of rats subjected to sham-EA at P5-6 (Figure 1A; *n* = 5). Heart rate was not affected by GD consistently. In contrast, EA applied at P5-6 significantly attenuated GD-induced pressor responses (Figure 1B; *P* < 0.05, *n* = 6). The vehicle (5% DMSO) administered into the rVLM did not influence BP responses to GD during sham-EA or EA (Figures 1A,B). However, EA-modulation of GD-induced pressor reflex responses was significantly reversed following administration of 8-SPT (a non-selective adenosine receptor antagonist) into the rVLM (Figure 1C; *P* < 0.05, *n* = 7).

### Specificity of Adenosine A_1_ and A_2__a_ Receptors in EA Inhibition of Reflex Elevations in BP

**FIGURE 2:**
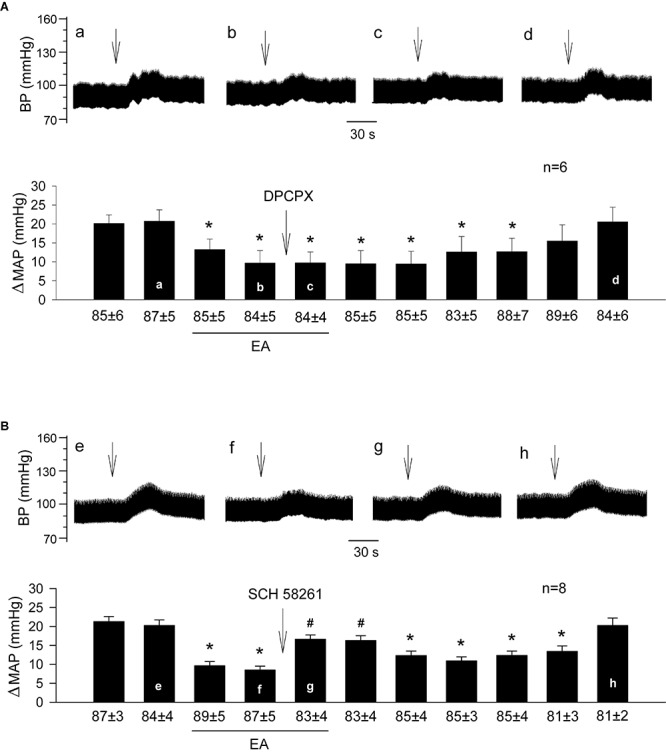
Influence of specific blockade of A_1_ or A_2a_ receptors in the rVLM on EA modulation of GD-induced pressor reflexes. Bars represent increases in mean arterial blood pressure (MAP) following GD. Values below each bar indicate the baseline MAP (means ± SE) before GD. **(A,B)** Microinjection of DPCPX (3 mM in 50 nl; an A_1_ receptor antagonist; **A**) or SCH 58261 (1 mM in 50 nl; an A_2a_ receptor antagonist; **B**) into the rVLM with EA treatment. ^∗^*P* < 0.05, a decrease of GD response after the onset of EA; ^#^*P* < 0.05, after vs. before microinjection into the rVLM. Labels a–d in **(A)** and e–h in **(B)** indicate examples of original BP tracings of a rat from each group; ↓, time of GD application. These data suggest that adenosine A_2a_, but not A_1_ receptors in the rVLM contribute to EA modulation of GD-induced pressor reflexes.

We observed that EA modulation of GD-induced pressor reflexes was not altered by DPCPX (an A_1_ receptor-specific inhibitor; Figure 2A, *n* = 6). However, it was reversed by rVLM microinjection of SCH 58261 (an adenosine A_2__a_ receptor-specific antagonist; Figure 2B, *n* = 8). The administration of the vehicle for both chemicals (5% DMSO) into the rVLM did not influence EA inhibitory effects on GD-induced elevations in BP, as demonstrated in Figure 1B.

### Changes in Pressor Reflexes After Activation or Inhibition of rVLM A_2__a_ Receptors in the Absence of EA Treatment

**FIGURE 3:**
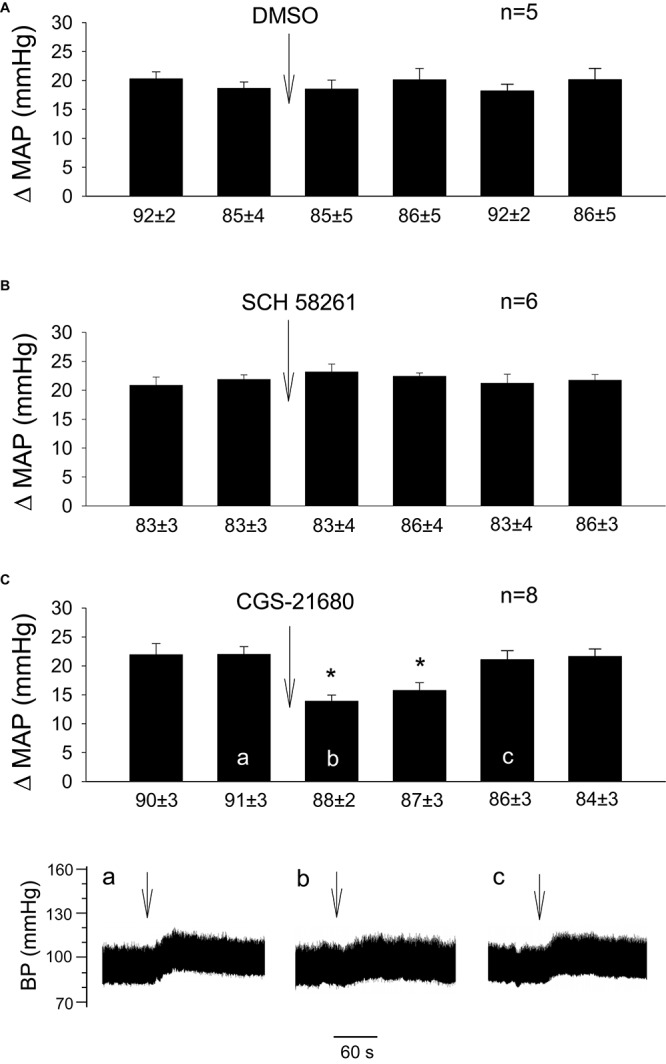
Influence of activation of A_2a_ receptors in the rVLM on GD-induced pressor reflexes. Bars represent increases in mean arterial blood pressure (MAP) following GD. Values below each bar indicate the baseline MAP (means ± SE) before GD. **(A–C)** Microinjection of 5% DMSO (50 nl; **A**), SCH 58261 (1 mM in 50 nl; an A_2a_ receptor antagonist; **B**) or CGS-21680 (0.4 mM in 50 nl; an A_2a_ receptor agonist; **C**) into the rVLM without EA treatment. ^∗^*P* < 0.05, after vs. before microinjection into the rVLM. Labels a–c in **(C)** indicate examples of the original BP tracings of a rat from this group; ↓, time of GD application. These data suggest that the activation of adenosine A_2a_ receptors in the rVLM contributes to inhibition of GD-induced pressor reflexes.

In the absence of EA, we observed consistent pressor responses to GD (Figure 3A). Further, we noted that GD-induced elevations in BP were unaltered by rVLM microinjection of 5% DMSO (the vehicle; Figure 3A; *n* = 5) or SCH 58261 (adenosine A_2__a_ receptor-selective antagonist; Figure 3B; *n* = 6). In contrast, microinjection of CGS-21680 (A_2__a_ receptor-selective agonist) into the rVLM significantly attenuated GD-induced pressor responses (Figure 3C; *P* < 0.05, *n* = 8), mimicking the EA’s inhibitory effect on pressor responses (Figure 1B).

### Involvement of Endogenous Opioids in Adenosine A_2__a_ Receptor-Mediated EA Modulation of Pressor Responses

**FIGURE 4:**
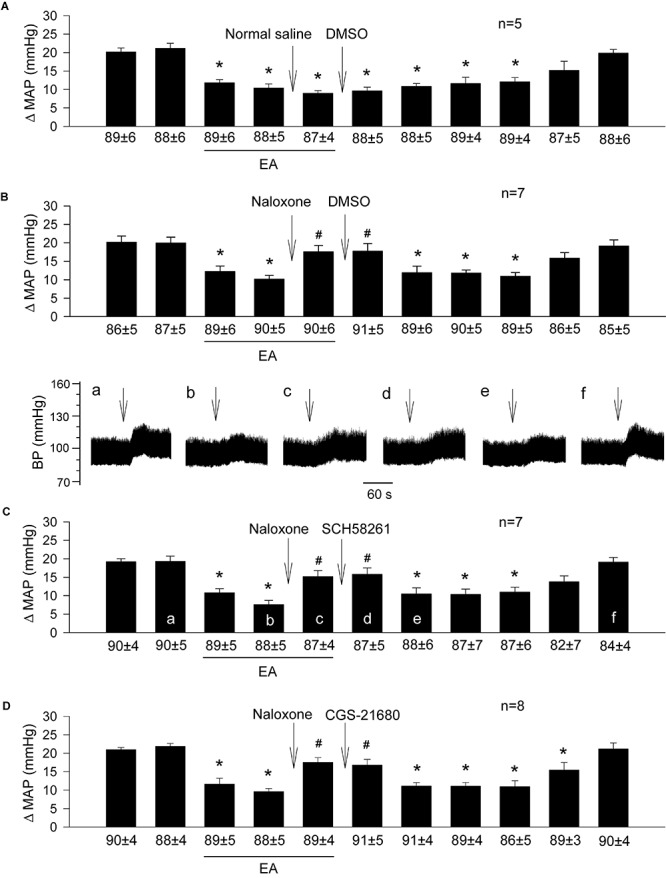
Importance of opioids in adenosine-mediated EA inhibition of GD-induced pressor reflexes through A_2a_ receptors in the rVLM. Bars represent increases in mean arterial blood pressure (MAP) following GD. Values below each bar indicate the baseline MAP (means ± SE) before GD. **(A)** Sequential microinjections of normal saline and 5% DMSO (both 50 nl) into the rVLM, which are vehicles for naloxone (a non-selective opioid receptor antagonist) and SCH 58261 & CGS-21680 (an A_2a_ receptor antagonist and agonist, correspondingly), respectively; **(B–D)** following microinjection of naloxone (1 mM in 50 nl) into the rVLM with EA treatment, 5% DMSO (the vehicle; **B**), SCH 58261 (1 mM in 50 nl; **C**) or CGS-21680 (0.4 mM in 50 nl; **D**) were administered into the same site of the rVLM. ^∗^*P* < 0.05, a decrease of GD response after the onset of EA; ^#^*P* < 0.05, after vs. before microinjection of naloxone into the rVLM. Labels a–f in **(C)** indicate examples of the original BP tracings of a rat from this group; ↓, time of GD application. Blockade or activation of A_2a_ receptors did not alter EA’s action in attenuating GD-evoked sympathoexcitatory cardiovascular reflexes after inhibition of opioid receptors in the rVLM. These data suggest that the involvement of rVLM adenosine A_2a_ receptors in EA modulation of GD-induced pressor reflexes is dependent on the presence of opioids.

Similar to the prior experiments in which rats underwent EA treatment (Figure 1B), we observed that 11-repeated GDs every 10 min induced consistent pressor responses despite two subsequent microinjections of 0.9% normal saline and 5% DMSO (the vehicles) into the rVLM (Figure 4A; *n* = 5). Microinjection of naloxone (a non-selective blocker of opioid receptors) into the rVLM significantly reversed EA inhibitory effects on GD-induced pressor responses (Figures 4B–D; all *P* < 0.05), lasting at least for 25 min. The naloxone-induced reversion of EA-modulated pressor responses was unchanged following subsequent administration of 5% DMSO (Figure 4B; *n* = 7), SCH 58261 (Figure 4C; *n* = 7) or CGS-21680 (Figure 4D; *n* = 8) into the same site of the rVLM, where the naloxone was employed.

### Histological Confirmation of Microinjection Sites

**FIGURE 5:**
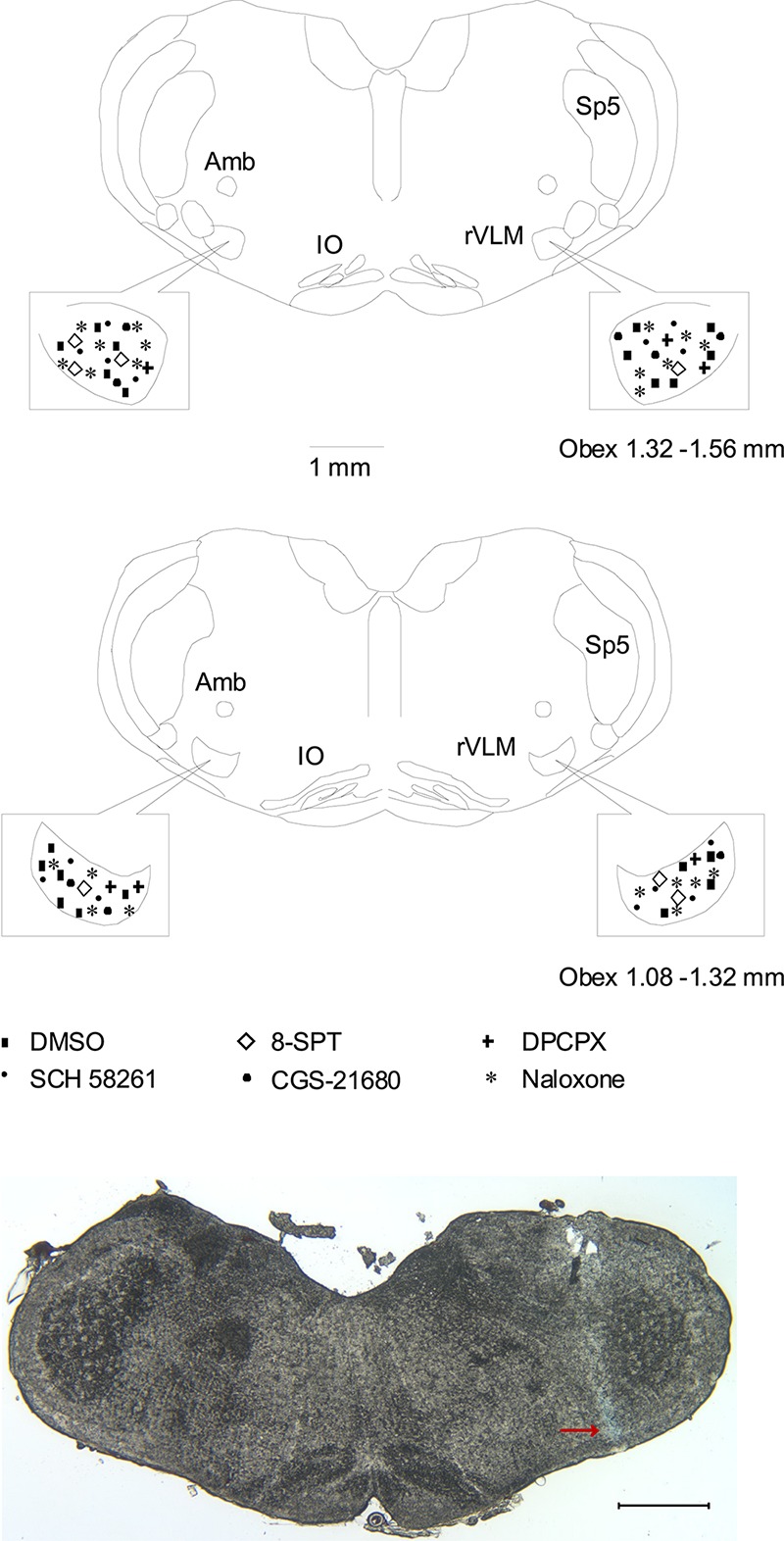
Anatomic locations of microinjection sites in the rat. **(Top two panels)** Composite maps displaying histologically verified sites of microinjections in the rVLM of rats. The box indicates the magnified area of the rVLM in these panels. Brain section shows the composite of planes of brain stem rostral to the obex (Paxinos and Watson’s atlas) (Paxinos and Watson, 2005). Symbols represent microinjection of 5% DMSO with and without normal saline (■), 8-SPT (⋄), DPCPX (+), SCH 58261 only (▲), CGS-21680 only (🌑), and naloxone (★). All the injections were unilateral (side chosen randomly). Sections are 1.08 – 1.32 and 1.32 – 1.56 mm rostral to the obex. Amb, nucleus ambiguous; IO, inferior olive; rVLM, rostral ventrolateral medulla; Sp5, spinal trigeminal nucleus. **(Bottom panel)** An original slide of the medulla oblongata (1.32 mm rostral to the obex) shows the blue-dyed track of a modified microdialysis probe insertion used for injection. Ventral aspect of blue dye represents the site of microinjection in the rVLM as indicated by an arrow. A scale bar represents 1 mm.

We examined brain slices of rats subjected to the microinjection into the rVLM and included animals in which the injections were verified to be located within the rVLM, as described above and shown in Figure 5. Microinjection sites were identified 1.8–2.3 mm lateral to the midline, 1.1–1.6 mm rostral to the obex, and 0.5–1.0 mm from the ventral surface. This region was consistent with the location of the rVLM, according to the atlas of Paxinos and Watson (2005).

### Co-localization of Adenosine A_2__a_ Receptors and Enkephalin in the rVLM

**FIGURE 6:**
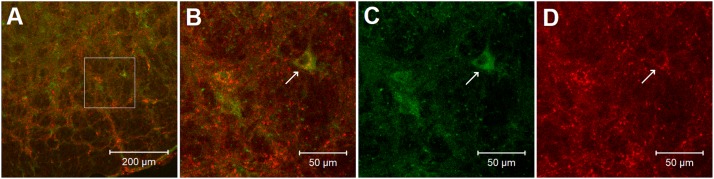
Confocal microscopic images show double-fluorescent labeling of adenosine A_2a_ receptors and enkephalin in the rVLM of a rat. **(A)** Low-power photomicrograph (1.44 mm rostral to the obex). **(B)** Magnified region displayed within the box in **(A)**. **(B)** Is a merged image from **(C,D)**. Arrows in **(B–D)** indicate a neuron co-labeled with adenosine A_2a_ receptors and enkephalin (yellow), adenosine A_2a_ receptors (green) and enkephalin (red), respectively. Scale bars in **(A,B–D)** represent 200 and 50 μm, correspondingly.

In the rVLM of all four rats, we found perikarya and neural fibers labeled with adenosine A_2__a_ receptors, and many neural fibers and some cell bodies stained with enkephalin. More importantly, we noted co-localization of adenosine A_2__a_ receptors with neurons containing enkephalin in these rats. Approximately 26 adenosine A_2__a_ receptor-containing neurons were identified in each section of the rat’s rVLM. Eighteen of these neurons were co-labeled with enkephalin. Thus, ∼70% of adenosine A_2__a_ receptor-containing neurons were enkephalinergic neurons. Figure 6 demonstrates confocal images showing the neuron double-labeled with adenosine A_2__a_ receptors and enkephalin in the rVLM of a rat.

## Discussion

Our findings show that underlying EA’s modulation of elevated BP is a complex mechanism involving both the purinergic and endogenous opioid systems in the rVLM. Blockade of adenosine receptors, specifically A_2__a_, but not A_1_ in the rVLM reversed EA modulation of GD-induced pressor responses. In the absence of EA, inhibition of adenosine A_2__a_ receptors in the rVLM did not alter pressor reflexes evoked by GD. However, the activation of these receptors attenuated the reflexes, mimicking EA’s inhibitory effect. Blockade of opioid receptors in the rVLM reversed EA’s action in inhibiting GD-induced pressor responses and blunted the contribution of A_2__a_ receptors to the mediation of the EA effect on reflexive responses as well. Moreover, neurons labeled with adenosine A_2__a_ receptors were co-localized with neurons stained with enkephalin in the rVLM. These data support our working hypothesis that adenosine, through rVLM A_2__a_, but not A_1__,_ receptor stimulation, contributes to the processing of EA-associated sympathoinhibition via an endogenous opioid mechanism.

We have previously shown that EA lowers elevated BP in experimental hypertensive models and patients with hypertension (Longhurst and Tjen-A-Looi, 2013; Li et al., 2015, 2016). Mechanical and chemical stimulation of visceral spinal afferents commonly causes reflex increases in BP due to enhanced sympathetic nerve activity (Li et al., 2002; Zhou et al., 2005b; Tjen-A-Looi et al., 2007) and the CNS is critically involved in the development of hypertension (Dampney, 1994). Our previous studies have demonstrated that EA at P5-6 attenuates reflex elevations in BP induced by visceral stimulation, like GD and chemical stimulation of gall bladder, compared to sham-EA without electrical stimulation and EA applied at non-acupoints. Our results indicate that the function of EA depends on both electrical activation and specific acupoints (Tjen-A-Looi et al., 2004, 2007; Zhou et al., 2005a; Guo et al., 2018). EA at P5-6 inhibits visceral sympathoexcitatory pressor responses through influence on multiple brain regions like arcuate nuclei, periaquductal gray, among others, which finally affect the rVLM and modulate hypertensive responses (Li and Longhurst, 2010). The rVLM is a crucial brain area that controls sympathetic outflow and integrates cardiovascular reflexes (Dampney, 1994). It also appears to serve as an essential area for processing the inhibitory effect of EA on sustained hypertension and pressor responses (Tjen-A-Looi et al., 2007; Li and Longhurst, 2010; Li et al., 2016). Visceral afferents including those located in the stomach pass through the splanchnic nerve to the brain regions, including the rVLM (Li et al., 2002; Zhou et al., 2005b; Li and Longhurst, 2010). Since EA at P5-6 inhibits evoked-activity of rVLM pre-sympathetic neurons during stimulation of the splanchnic nerve (Zhou et al., 2005b; Tjen-A-Looi et al., 2007), excitatory cardiovascular responses induced by splanchnic inputs to the rVLM likely are inhibited by EA through stimulation of somatic afferents.

We observed previously that EA modulates pressor cardiovascular responses induced by visceral stimulation through several neurotransmitters or neuromodulators, including opioids, GABA, nociception, and serotonin in the rVLM (Tjen-A-Looi et al., 2007; Moazzami et al., 2010). However, inhibition of any one of these neural substances in the rVLM only partially eliminates EA’s hypotensive effect, implying the contribution of other modulators during EA. In this regard, adenosine is released during neuronal activation (Spyer et al., 1997; St Lambert et al., 1997; Burnstock, 2007) and primarily modulates neuronal function through P1 receptors (Spyer et al., 1997; Burnstock, 2007). Adenosine receptors are distributed in various regions in CNS, including the rVLM (Thomas and Spyer, 1999; Nassar and bdel-Rahman, 2008, 2009; Jiang et al., 2011). Moreover, there is evidence showing that adenosine modulates rVLM activity (Thomas and Spyer, 1999). Although adenosine likely serves a peripheral local role in acupuncture antinociception (Goldman et al., 2010), its contribution to modulatory action of acupuncture in the brain, particularly in the rVLM, is uncertain. In the present study, we found that non-selective inhibition of adenosine receptors in the rVLM reversed the EA-inhibitory effect on GD-induced pressor responses, implying involvement of adenosine and its receptors in processing EA’s action in the rVLM.

Adenosine P1 receptors are sub-classified into four G protein-coupled receptors: A_1_, A_2__a_, A_2__b_, and A_3_. The expression of patterns of these receptors varies among cell types, and adenosine induces a multitude of physiological effects, including neuronal activity in the body. While all four adenosine receptor subtypes are present in CNS, the adenosine A_1_ and A_2__a_ receptors are predominantly expressed in the brain (Ribeiro et al., 2002; Borea et al., 2018). Previous studies showed that selective pharmacological activation of A_1_ or A_2__a_ receptors in the rVLM induces pressor or depressor responses, respectively (Nassar and bdel-Rahman, 2008, 2009; Jiang et al., 2011). Based on these previous studies, we hypothesized that adenosine receptors A_2__a_ rather than A_1_ are involved in EA’s inhibitory effect on excitatory cardiovascular responses. Data from the present study support our working hypothesis, which demonstrated that specific inhibition of A_2__a_, but not A_1_ receptors in the rVLM reversed EA modulation of GD-induced pressor reflexes. Furthermore, the application of the specific A_2__a_ receptor agonist into the rVLM reduces GD-induced pressor responses without EA treatment, which mimics EA’s effect. Blockade of A_2__a_ receptors in the rVLM did not alter GD-induced pressor reflexes in the absence of EA, which implies no involvement of this type of adenosine receptors in primary GD-induced excitatory cardiovascular responses. Our results support the notion about the role of adenosine via A_2__a_ receptors in the rVLM in processing EA’s modulatory action on visceral sympathoexcitatory reflexes. Goldman and colleagues showed that adenosine A_1_ receptors participate in antinociceptive effects of manual acupuncture in peripheral tissues (Goldman et al., 2010), but they unlikely contribute to EA modulation of cardiovascular pressor responses in the rVLM as A_2__a_ receptors do. As such, we suggest that adenosine may contribute to the effect of acupuncture through different types of adenosine receptors in CNS versus peripheral tissues. Also, different kinds of adenosine receptors may be involved in acupuncture’s action in various physiological and pathophysiological statuses as well as during the application of varying acupuncture stimulation (e.g., EA vs. manual acupuncture). Addressing these issues requires further investigations.

In general, adenosine A_1_ receptors are coupled to G_*i/o*_ and A_2__a_ receptors to G_s_ protein. Thus, adenosine triggers opposite effects on adenylate cyclase and its associated cAMP intracellular pathway, leading to inhibition and excitation of cells through adenosine A_1_ and A_2__a_ receptors respectively (Ribeiro et al., 2002; Borea et al., 2018). Excitation of pre-sympathetic premotor neurons in the rVLM usually leads to an increase in BP, while inhibition of them causes a decrease in BP (Dampney, 1994). It seems to be reasonable to assume that activation of A_1_ and A_2__a_ receptors in the rVLM premotor neurons contributes to reduction and elevation in BP, accordingly. However, several lines of evidence show that selective pharmacological excitation of adenosine A_1_ or A_2__a_ receptors in the rVLM leads to increase or decrease in BP, respectively (Nassar and bdel-Rahman, 2008, 2009; Jiang et al., 2011), which disagree with the assumption aforementioned. These reports imply that adenosine unlikely influences post-synaptic premotor neurons in rVLM through A_1_ or A_2__a_ receptors directly. In this respect, it is possible that adenosine may, through these subtypes, modulate the release of neurotransmitters or modulators from pre-synaptic neurons, which then affect rVLM premotor neurons and BP. Previous studies in the brain support this possibility, demonstrating that A_1_ and A_2__a_ receptors are involved in inhibition and enhancement of pre-synaptic release of neurotransmitters (Cunha and Ribeiro, 2000; Ribeiro et al., 2002; Scislo and O’Leary, 2006). In particular, adenosine reduces GABA release through pre-synaptic A_1_ receptors in the rVLM, which contributes to increasing the rVLM activity and elevated BP during stimulation of the hypothalamic defense area (Thomas and Spyer, 1996, 1999; St Lambert et al., 1996; Han et al., 2011). Given our previous findings of EA’s inhibitory effects on pressor cardiovascular responses through GABA, opioids, and nociceptin in the rVLM (Crisostomo et al., 2005; Tjen-A-Looi et al., 2007), we speculate that EA likely enhances these neurotransmitters or modulators through adenosine-mediated A_2__a_ receptor mechanisms in the rVLM and lowers elevated BP during cardiovascular pressor responses.

**FIGURE 7:**
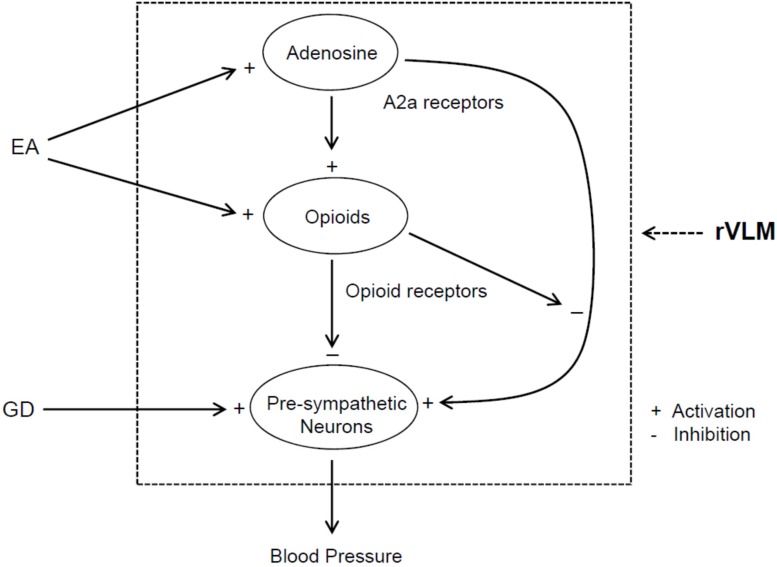
Potential neural pathways associated with the action of adenosine A_2a_ receptor-opioids in the rVLM in EA regulation of sympathetic outflow and cardiovascular pressor responses during visceral stimulation. +, indicates activation or enhancement of the system; –, indicates inhibition of the system; EA, electroacupuncture; GD, gastric distension. EA likely activates adenosine A_2a_ receptors in the rVLM, which promotes opioid generation. The activation of opioid receptors causes inhibition of the pre-sympathetic activity of rVLM neurons directly as well as through suppression of A_2a_ receptor-mediated excitation of these neurons, leading to a reduction in blood pressure.

A body of evidence shows interactions between the adenosine and opioid systems in CNS (Ribeiro et al., 2002; Borea et al., 2018). In this regard, a previous study notably demonstrated that antinociceptive effects of intracerebroventricular administration of A_2__a_ receptor agonist are regulated, at least in part, through an opioid mechanism (By et al., 2011). Opioids play a role in EA regulation of cardiovascular responses (Tjen-A-Looi et al., 2007; Li and Longhurst, 2010). In this respect, EA reduces rVLM pre-sympathetic activity and reflex-induced elevations in BP through an opioid mechanism involving enkephalins (via δ- and μ-receptors) and endorphins (μ-receptors), but not dynorphins (κ-receptors) (Li et al., 2001, 2002; Tjen-A-Looi et al., 2007). Moreover, administering one of δ- and μ-receptor agonists into the rVLM attenuates pressor responses caused by visceral stimulation, including GD (Li et al., 2002, 2013; Crisostomo et al., 2005; Tjen-A-Looi et al., 2007), which mimics EA’s effect. Enkephalins are synthesized in EA-activated rVLM neurons (Guo et al., 2004), while endorphins are produced in the hypothalamic arcuate nucleus and then transported to the rVLM (Li et al., 2009). Preproenkephalin mRNA in the rVLM is increased following single and repetitive EA in normotensive models (Li et al., 2010, 2012). It is unknown if opioids are involved in adenosine regulation of elevated BP during EA. In the present study, we observed that non-selective blockade of opioid receptors in the rVLM partially reverses EA inhibitory effects on GD-induced pressor responses, which are consistent with our previous findings of the importance of opioids in the EA’s impact, as aforementioned (Li et al., 2002, 2013). More interestingly, we noted that neither inhibition nor activation of A_2__a_ receptors in the rVLM cause any changes in GD-induced elevation in BP following blockade of opioid receptors in animals subjected to EA. These observations suggest that the involvement of rVLM adenosine A_2__a_ receptors in EA modulation of GD-induced pressor reflexes is, at least in part, specifically dependent on the presence of endogenous opioids. Furthermore, our anatomical data demonstrate that A_2__*a*_ receptors in the rVLM co-localize with enkephalin-containing neurons, indicating an influence of adenosine on these enkephalinergic neurons through A_2__a_ receptors in the rVLM. With our previous and current findings taken together, we think that EA likely excites rVLM enkephalin-containing neurons, at least in part, through adenosine via A_2__a_ receptors and promotes enkephalin release, leading to modulation of the pre-sympathetic activity of rVLM neurons and elevated BP evoked by GD (Figure 7).

Adenosine A_2__a_ receptors also are present in the post-synaptic neurons, including rVLM premotor neurons (Thomas and Spyer, 1999; Ribeiro et al., 2002; Borea et al., 2018). Thus, we cannot exclude a possibility that A_2__a_ receptors in these neurons are activated during EA. However, the potential excitatory effect of activation of A_2__a_ receptors on the post-synaptic neurons might be counteracted by the inhibitory action, resulting from activation of adenosine A_1_ receptors as well as receptors associated with other neurotransmitters or neuromodulators like opioids, GABA, and among others. In this respect, particularly, there is evidence showing that δ-receptor agonists inhibit A_2__a_ receptor-mediated activation of adenylyl cyclase, leading to a reduction in cAMP-regulated phosphorylation of neurons in the striatum and caudate putamen of rats (Noble and Cox, 1995; By et al., 2011). Further studies are needed to examine this possibility (Figure 7).

One limitation of the present study is that we cannot identify directly the source of EA-activated neurons that generate adenosine due to technical restrictions. However, as we demonstrated previously, EA activates neurons in multiple brain regions like the paraventricular nucleus, arcuate nucleus, nucleus tractus solilatrii, caudal ventrolateral medulla, and raphe nucleus, which project to the rVLM directly with mono-synaptic axons (Li et al., 2009; Moazzami et al., 2010; Tjen-A-Looi et al., 2013, 2016; Guo and Malik, 2019). As such, it is reasonable to suggest that neurons generating adenosine during EA may be present in the rVLM as well as in other brain areas that have direct connections with the rVLM.

*Perspective and Significance* Elevated BP during sympathoexcitatory responses frequently increase morbidity and mortality of patients with cardiovascular diseases (Guo et al., 2009; Li and Longhurst, 2010). Acupuncture’s effects on reflex-induced elevations in BP provide important clues to clinical treatment of acute pressor responses and essential hypertension (Li and Longhurst, 2010; Longhurst and Tjen-A-Looi, 2013). In this respect, using modalities of stimulation proven to reduce elevated BP in reflex animal models effectively, we have found in patients with essential hypertension that repetitive EA lowers BP for weeks following therapy (Li and Longhurst, 2010; Li et al., 2015). The acute reflex studies in anesthetized animals have allowed in-depth mechanistic investigation of EA-related alterations in CNS processing of autonomic outflow to complement studies in sustained hypertension. The present study identified adenosine’s role in the rVLM through opioids during EA modulation of reflex-induced elevations in BP. Our results thus expand the understanding of neural mechanisms underlying EA inhibition of elevated BP and suggest conducting further studies to elucidate these mechanisms further. With this respect, we may examine if adenosine receptors in other brain regions participate in EA modulation of cardiovascular responses. Also, it will be interesting to test if adenosine via specific receptors interacts with neurotransmitters or neuromodulators other than opioids in the brain areas during EA’s effects on elevated BP, including through adenosine receptor heteromers. These proposed studies will further explore central mechanisms underlying the specific contribution of adenosine to EA’s action in regulating cardiovascular responses.

Caffeine is a non-specific antagonist for adenosine receptors (Ribeiro et al., 2002). In light of our new results, patients with hypertension and other cardiovascular diseases may need to avoid consuming high levels of caffeine, when they undergo acupuncture treatment to achieve the full benefits of this therapy. These stimulants may reduce the activity of adenosine A_2__a_ receptors and thus the beneficial effects of acupuncture. On the other hand, our result also suggests that an agonist drug, like deoxycoformycin (Pentostatin) that can increase adenosine may act through the adenosine A_2__a_ receptors to enhance the clinical benefits of acupuncture, particularly, in management of cardiovascular disorders.

## Conclusion

In conclusion, the results from the present study demonstrate that A_2__a_ adenosine receptors in the rVLM play a role in central processing EA’s action in modulating reflex excitatory cardiovascular responses. The participation of rVLM adenosine A_2__*a*_ receptors in EA modulation of GD-induced pressor reflexes is, at least in part, dependent on the presence of opioids. These new findings extend our knowledge regarding central neural mechanisms underlying acupuncture’s influence on cardiovascular function and provide hints at possible ways to enhance the clinical response of acupuncture therapy.

## Data Availability Statement

The datasets generated for this study are available on request to the corresponding author.

## Ethics Statement

The animal study was reviewed and approved by the Animal Care and Use Committee of the University of California, Irvine.

## Author Contributions

Z-LG and SM responsible for the conception and design of research, and wrote the manuscript. Z-LG and TS performed the experiments. All authors analyzed and interpreted the results of the experimental data, and edited, revised, and approved the final version of the manuscript. Z-LG prepared the figures.

## Conflict of Interest

The authors declare that the research was conducted in the absence of any commercial or financial relationships that could be construed as a potential conflict of interest.

## Footnotes

1 www.Amscan.com

## Abbreviations

BP: blood pressure
CNS: central nervous system
DMSO: dimethyl sulfoxide
EA: electroacupuncture
GD: gastric distension
MAP: mean arterial blood pressure
rVLM: rostral ventrolateral medulla.
